# Supplementary material for: Tribological and Heat Transfer Investigation of Graphene Oxide Coatings on Nylon Rotating Bands in an Artillery System
Source: Nanomaterials (Basel). 2024 Dec 3;14(23):1943. doi: 10.3390/nano14231943 (PMC11643560; doi:10.3390/nano14231943)
Supplement: Supplementary file 1 [file nanomaterials-14-01943-s001.zip › nanomaterials-3311526-supplementary.pdf]

Tribological and heat transfer investigation of graphene oxide coatings on nylon rotating band in artillery system

Hongbin Chen [1]*, Zeyang Meng [1],Shuang Yi [1]

[1]School of Mechanical Engineering, Nanjing University of Science and Technology, 210094, China

Corresponding author:

*To whom all correspondence should be addressed.

Hongbin Chen, E-mail:chb12013017@njust.edu.cn

1.Thermographic images under different external loads.

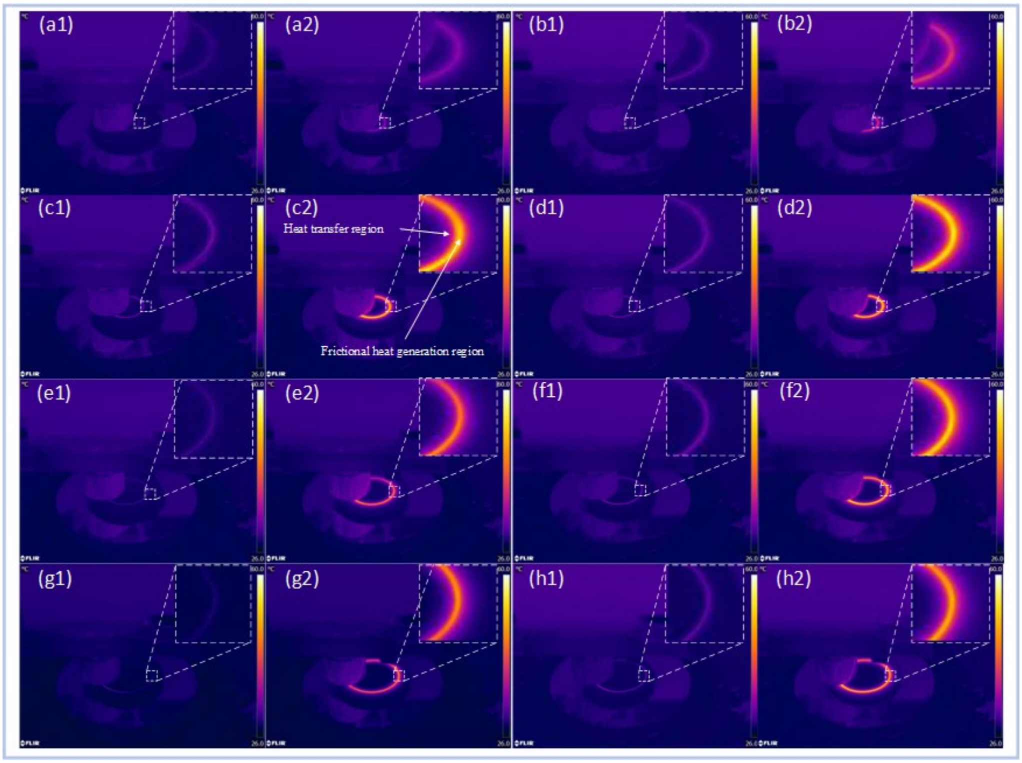

Figure S1. The changes of surface temperature under four types of loads. (a) Thermal images of the Nylon specimen under an external load of 4 N after rotating for 1s and 20 s. (b) Thermal images of the GO-Nylon specimen under an external load of 4 N after rotating for 1s and 20 s. (c) Thermal images of the Nylon specimen under an external load of 8 N after rotating for 1s and 20 s. (d) Thermal images of the GO-Nylon specimen under an external load of 8 N after rotating for 1s and 20 s. (e) Thermal images of the Nylon specimen under an external load of 12 N after rotating for 1s and 20 s. (f) Thermal images of the GO-Nylon specimen under an external load of 12 N after rotating for 1s and 20 s. (g) Thermal images of the Nylon specimen under an external load of 15 N after rotating for 1 s and 20 s. (h) Thermal images of the GO-Nylon specimen under an external load of 15 N after rotating for 1 s and 20 s.

2. Morphological changes of the specimens under an optical microscope before and after coating preparation.

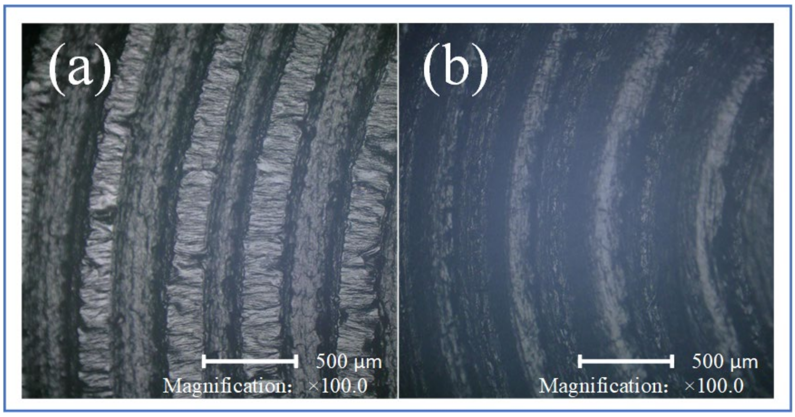

Figure S2 States of the lower specimen before and after the prefabrication of the graphite oxide surface. (a) Optical microscope image of the surface of the lower specimen with a surface roughness of Ra 3.2 after the preparation of the graphene oxide coating. (b) Optical microscope image of the original surface of the lower specimen.

3. Raman spectra of the surface materials of the upper specimen after friction.

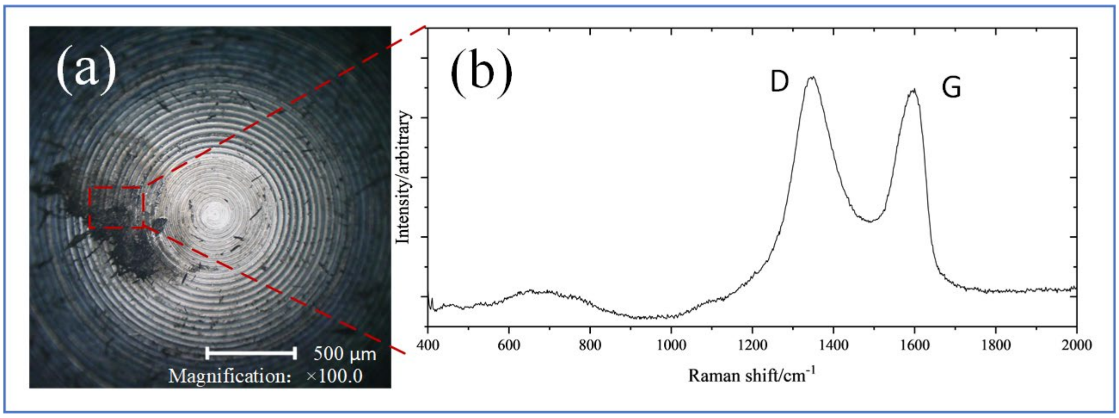

Figure S3 Results of Raman spectra on the surface of the upper specimen after friction. (a) Optical microscope image of the wear mark position on the upper specimen when the surface roughness is Ra 6.3 and the external load is 12 N. (b) Seleted area of Raman spectra measurement results in (a), which displayed graphene oxide specific peak D and G on 1354 cm-1 and 1586 cm-1, respectively.
